# Virulence and molecular genetic diversity, variation, and evolution of the *Puccinia triticina* population in Hebei Province of China from 2001 to 2010

**DOI:** 10.3389/fpls.2023.1095677

**Published:** 2023-03-06

**Authors:** Lin Zhang, Linya Zhang, Qingfang Meng, Hongfei Yan, Daqun Liu

**Affiliations:** ^1^ College of Plant Protection, Hebei Agricultural University, Technological Innovation Center for Biological Control of Crop Diseases and Insect Pests of Hebei Province, Baoding, China; ^2^ School of Landscape and Ecological Engineering, Hebei Engineering University, Handan, China

**Keywords:** *Puccinia triticina*, race, virulence, EST-SSR, genetic diversity

## Abstract

Wheat leaf rust, caused by *Puccinia triticina*, is one of the most important fungal diseases of wheat in China. However, little is known about the dynamic changes of population structure and genetic diversity of *P. triticina* during a period of time. In this study, 247 isolates of *P. triticina* collected from Hebei Province from 2001 to 2010 were tested on 36 Thatcher near-isogenic lines for virulence diversity and detected by 21 pairs of Expressed Sequence Tag derived Simple Sequence Repeat (EST-SSR) primers for genetic diversity. A total of 204 isolates were successfully identified as 164 races, and THTT, THST, PHRT, THTS, and PHTT were the most common races in Hebei Province from 2001 to 2010. The cluster analysis based on virulence showed that *P. triticina* has a rich virulence polymorphism, which had a certain correlation with the years, while the cluster analysis based on EST-SSR showed that the genetic diversity of the *P. triticina* population was significantly different between years in Hebei Province from 2001 to 2010. In addition, the population structure of *P. triticina* may have changed greatly in 2007 and 2009, which was significantly different from that of 2001–2006 on either virulence or genetic characteristics. The variation frequency of the population structure had an increasing trend during this period. From 2001 to 2010, there was a certain degree of gene flow among the *P. triticina* populations. No significant correlation was found between virulence and molecular polymorphism. The genetic differentiation analysis of the 10 tested populations (each year as a population) showed that the coefficient of genetic differentiation (Gst) was 0.27, indicating that there was a certain genetic differentiation among or within populations of *P. triticina* in Hebei Province. The genetic variation within populations (73.08%) was higher than that among populations (26.92%), which indicated that the genetic variations were mainly found within populations. Our study provides the foundation for a better understanding of the population structure change and genetic diversity of *P. triticina* over a period in Hebei Province of China.

## Introduction

Wheat leaf rust, caused by *Puccinia triticina* Erikss., is the most common fungal disease of common wheat (*Triticum aestivum* L.) in major wheat-growing regions worldwide (Bolton et al., 2008). The occurrence and epidemic of leaf rust have seriously affected the yield and quality of wheat, and it is one of the main factors affecting global wheat production (Huerta-Espino et al., 2011). Due to the difference in wheat cultivars and disease period, the yield loss may be 7%–30%, even more than 50% in severe cases (Huerta-Espino et al., 2011; Zhang et al., 2022). China is the world’s largest wheat-producing country, with more than 23 million hectares of wheat-planting area and more than 138 million tons yield in 2022 (USDA-FAS). A serious incidence of leaf rust with severe epidemics in 1969, 1973, 1975, 1979, 2012, 2013, and 2015 caused significant yield losses in major wheat-producing regions in China (Zhang et al., 2020a; Zhang et al., 2020b). Especially in 2015, wheat leaf rust was widespread in Hebei, Shandong, Henan, Jiangsu, Anhui, Hubei, and other provinces of China (Zhang et al., 2015; Zhang et al., 2020a; Zhang et al., 2020b; Zhang et al., 2022). Hebei Province is one of the main wheat-producing regions in China, and wheat in this region is affected by leaf rust every year. The promotion and application of resistant cultivars are the most economical, effective, and environment-friendly methods to control the disease (Pink, 2002). However, *P. triticina* has a wide range of temperature tolerance and highly variable virulence. These features easily lead to the loss of the effectiveness of leaf rust resistance genes, which makes leaf rust difficult to control. Therefore, it is of great significance to understand the population virulence and genetic structure of *P. triticina* for controlling the disease and providing guidance for breeders in wheat resistance breeding programs.

In order to monitor the virulence dynamics and genetic diversity of *P. triticina*, studies on race surveys and virulence identification of *P. triticina* have been carried out in different main wheat-producing regions worldwide since the 1920s. The annual survey of race and virulence of *P. triticina* in the United States began in 1926 (Mains and Jackson, 1926; Johnston et al., 1968). Kolmer and Fajolu (2022) conducted a comprehensive and systematic analysis of the race and virulence dynamics of *P. triticina* in the United States from 2000 to 2020. Similar surveys and studies have been carried out in Canada since 1931 and from 1997 to 2010 (Johnson, 1956; Kolmer, 1991; Kolmer, 1999, Kolmer, 2001; McCallum et al., 2016). Moreover, similar studies have also been carried out in France (Goyeau et al., 2006), South America (Germán et al., 2007), Ukraine (Elyasi-Gomari and Panteleev, 2006), India (Bhardwaj et al., 2010; Bhardwaj et al., 2019), and Egypt (El-Orabey et al., 2018). Wang (1947) first reported three Chinese *P. triticina* races that were identified based on eight international identification wheat cultivars chosen by Johnston and Mains (1932). Then, based on the Thatcher near-isogenic lines, the race and virulence studies of *P. triticina* since 1986 were carried out in China (Chen et al., 1993; Chen et al., 1998; Hu and Roelfs, 1989; Yang et al., 2004; Liu and Chen, 2012; Kolmer, 2015; Zhang et al., 2020a; Zhang et al., 2020b). Investigations of races and virulence of *P. triticina* can promote the study of genetic structure and virulence variation of *P. triticina* populations.

In addition to the methods of the race and virulence identification of *P. triticina*, many molecular marker technologies have also been applied to the genetic diversity analysis of *P. triticina* populations in recent years. Park et al. (2000) analyzed the population structure of *P. triticina* in Western Europe by assessing the variability in pathogenicity and randomly amplified polymorphic DNA (RAPD) among 61 single-uredinial isolates, and little evidence was found for robust distinct clusters among the isolates. In 2001, Kolmer (2001) found a correlation between virulence phenotype and amplified fragment length polymorphism (AFLP) genotype for 69 isolates of *P. triticina* from Canada. In 2007, Kolmer and Ordoñez (2007) identified and analyzed the virulence and molecular genotype of isolates of *P. triticina* collected from common wheat in Central Asia and the Caucasus regions based on near-isogenic lines of Thatcher and SSR molecular markers and found that all populations of *P. triticina* from Central Asia and the Caucasus regions were significantly differentiated from that of North America. In addition, Zhang et al. (2015) reported a high genetic diversity in *P. triticina* populations collected in Hebei Province of China based on universally primed PCR (UP-PCR) primers. The Expressed Sequence Tag derived Simple Sequence Repeat (EST-SSR) markers of *P. triticina* have been developed and used in genetic diversity analysis for *P. triticina* in Canada and China (Wang et al., 2010a; Wang et al., 2010b; Zhao et al., 2014; Zhang et al., 2018). The EST-SSRs were polymorphic and informative in determining intraspecific genetic diversity (Wang et al. 2010b). *P. triticina* isolates collected from Hebei Province of China in 2008 and 2010 showed high diversity and differences between two populations on EST-SSR (Zhang et al., 2018). However, it only revealed the population differences between 2 years in Hebei Province, which did not fully reveal the *P. triticina* population dynamic patterns in this region. In this study, we analyzed the population genetic diversity of *P. triticina* in Hebei Province for 10 years (2001–2010) based on the results of Zhang et al. (2018), which can provide a basis for further exploring the race variation patterns of *P. triticina* and the molecular mechanism of race virulence evolution.

## Materials and methods

### Leaf rust sample collection and multiplication

**Figure 1:**
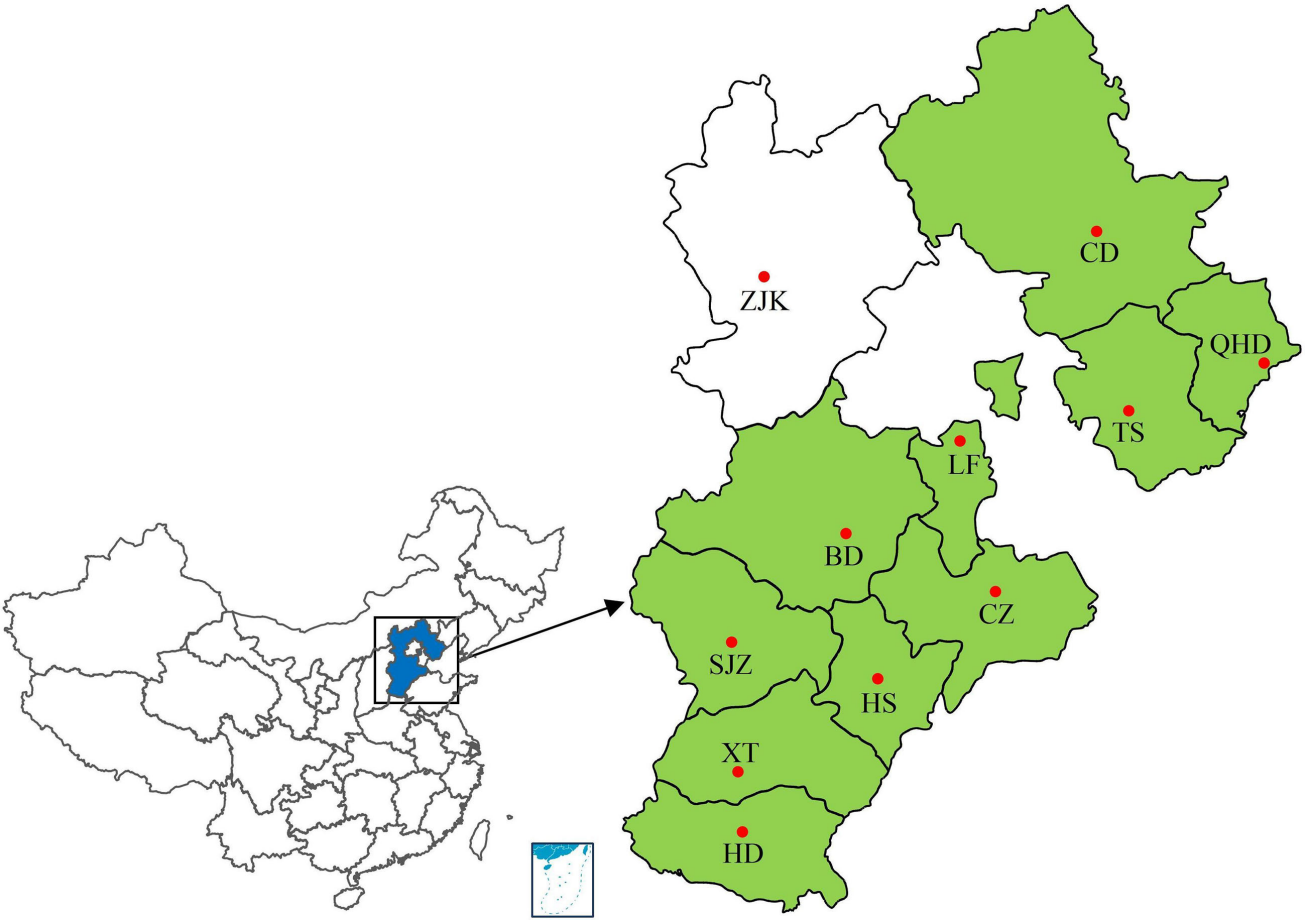
Collection sites of wheat leaf rust samples from 2001 to 2010. Colored regions represent regions of sample sources. SJZ, Shijiazhuang; BD, Baoding; LF, Langfang; CZ, Cangzhou; HS, Hengshui; TS, Tangshan; CD, Chengde; XT, Xingtai; HD, Handan; QHD, Qinghuangdao. •, location of provincial capital.

Leaf rust samples were collected from 10 regions of Hebei Province from 2001 to 2010 during the wheat filling period by researchers from the College of Plant Protection, Hebei Agricultural University (**Figure 1**; **Supplementary Table S1**). All samples were collected from fields under natural infection. Wheat leaves with uredinia of *P. triticina* sampled from a single plant or cultivar were treated as a single sample. The sample treatment and single-uredinial isolate multiplication were performed as described by Zhang et al. (2020a). Urediniospores collected from each sample were inoculated to the highly susceptible cultivar ‘Zhengzhou 5389’ to obtain a single uredinium. Urediniospores from the single uredinium were increased by inoculating the new seedlings of ‘Zhengzhou 5389’ using the same inoculation procedure. Approximately 10–14 days after inoculation, urediniospores were collected by shaking the leaf, put into glass tubes for drying and lyophilization, and stored at -20°C.

### Race and virulence phenotype data

All of the race and virulence phenotype data were conducted and stored in the Centre of Wheat Rust Research, Hebei Agricultural University. Race identification and virulence determination of isolates were performed as described by Zhang et al. (2020a). Urediniospores collected from each sample were inoculated on two sets of Thatcher near-isogenic lines, which included 16 standardized differentials (with single resistance genes *Lr1*, *Lr2a*, *Lr2c*, *Lr3*; *Lr9*, *Lr16*, *Lr24*, *Lr26*; *Lr3Ka*, *Lr11*, *Lr17*, *Lr30*; *LrB*, *Lr10*, *Lr14a*, and *Lr18*) and 20 extra differentials (with resistance genes *Lr2b*, *Lr3bg*, *Lr14b*, *Lr15*, *Lr19*, *Lr21*, *Lr23*, *Lr25*, *Lr28*, *Lr29*, *Lr32*, *Lr33*, *Lr33+34*, *Lr36*, *Lr37*, *Lr38*, *Lr39*, *Lr42*, *Lr44*, and *Lr45*). The susceptible Thatcher was used as a control. When uredinia were fully developed on the leaves of Thatcher approximately 12 days post-inoculation (dpi), infection types (ITs) on the near-isogenic lines were recorded based on a 0–4 scale (Roelfs, 1984). According to the ITs of the tested isolates on 16 standardized differentials, races were determined on the basis of a 4-letter code as outlined by Kolmer (2001).

### DNA extraction and PCR amplification

The genomic DNA of each isolate sample was extracted from the urediniospores according to the modified Cetyltrimethylammonium Bromide (CTAB) method described by Gao (2003). The 21 pairs of EST-SSR primers (**Supplementary Table S2**) developed by Wang et al. (2010a, 2010b) were used for PCR reaction, and the total reaction system and amplification procedure were performed as described by Zhang et al. (2018). PCR products were separated by 10% (w/v) polyacrylamide gel in 0.5× TBE buffer (45 mM Tris, 45 mM boric acid, and 1 mM EDTA), visualized by silver staining.

### Data analysis

To analyze the genetic relationships among selected *P. triticina* isolates, two binary matrices based on virulence ITs and EST-SSR data were constructed respectively as described by Zhang et al. (2018). Briefly, the virulence binary matrix of 0 and 1 was constructed based on the high (1) or low (0) ITs, and the EST-SSR binary matrix of 0 and 1 was generated based on the presence (1) or absence (0) of PCR amplification bands. The software program NTSYS-pc version 2.10 (Rohlf, 2000) was used to construct the dendrograms, the two-dimensional (2D) principal coordinates analysis (PCA), and the correlation between the virulence and EST-SSR. The software POPGENE version 1.32 (Yeh et al., 1999) was used to assess the population diversity of *P. triticina*. To evaluate population genetic differentiations, the analysis of molecular variance (AMOVA) was performed using the AMOVA model of Arlequin3.ll software (http://cmpg.unibe.ch/software/arlequin3).

## Results

### Dynamics of races

In total, 247 single-uredinial isolates were selected from Hebei Province from 2001 to 2010, of which 204 isolates were successfully identified as 164 races (**Table 1**; **Supplementary Table S1**). THTT, THST, PHRT, THTS, and PHTT were the predominant races, with frequencies of 7.35%, 5.39%, 4.41%, 4.41%, and 2.45%, respectively.

**Table 1:** Races and number of isolates determined each year from 2001 to 2010.

Year	Number of isolates	Number of isolates identified	Number of races	Races
2001	23	9	7	PHJN*(2), THJQ(2), PCTF, PHJG, PHJS*, PHSS*, THHT
2002	24	14	13	PHJS*(2), DHGK, FGGT, FHGS, FHJQ, FHQS, LHGS, PGBT, PGJN, PHJN*, PHJQ, PHSQ, PHSS*
2003	6	6	6	FHJS, FHKS, FHSS*, KHTS, PHLS, PHST
2004	27	27	27	DHQS, FCJT, LBGL, MHGN, NHJN, PCBL, PCDN, PCGT, PCJD, PCJN, PCQS, PGPN, PHBL, PHDN, PHGP, PHGR, PHGS, PHGT, PHJN*, PHQB, PHQP, PHQT, PHRT, PHSN, THCT, THHP, THQS
2005	20	19	18	PHRT(2), CCQS, DBGN, FGJS, FHBQ, FHBS, FHDQ, FHGQ, FHHT, FHQS, FHSS, FHTS, PHBL, PHJS*, PHQT, PHSS, TCTR, THGS
2006	28	14	12	FCHT(2), FCRT(2), FBKT, FCGT, FCHS, FCKT, FCTT, FFTT, FHKT*, PBHP, RFDT, THNT
2007	30	26	19	THST*(4), PHRT*(3), PHHP(2), PHSN(2), FGQT, MGJS, MHTS, PCNL, PCTT, PHJT, SHRT, TCLT, THCT, THFT, THHT, THLT, THRT, THSQ, THTT*
2008	30	30	24	THTT*(3), PHTT*(3), THST(2), THKT*(2), MHGT, PCCT, PCHP, PCKT, PCQT, PCRT, PFMP, PHBT, PHDT, PHGS, PHQP, PHRT*, PHST*, SHRT, TCHT, THBN, THJT*, THLP, THNS, THSS
2009	30	30	18	THTT*(8), THTS*(5), PHTT*(2), KHTS, PCJT, PHGR, PHJT, PHKP, PHRT, PHTM, PHTN, PHTS, TGTP, THHS, THKP, THKT, THPT, THST,
2010	29	29	20	THST*(4), THTS*(4), THTT*(3), THRT(2), MHST, PCRT, PCTT, PHHS, PHRT, PHSN, PHSS, PHST, PHTN, THFK, THHT, THKT, THQS, THTN, THTP, THTR
Total	247	204	164	

The number in parentheses is the number of races. “*”: The predominant race of each year.

### Virulence polymorphism analysis

The similarity coefficient of virulence cluster analysis between 204 isolates was 0.53–0.98 by constructing dendrograms of virulence based on Unweighted Pair-Group Method with Arithmetic (UPGMA) method (**Supplementary Figure S1**). All isolates were clustered into 13 groups at a similarity coefficient of 0.685. The 187 isolates (91.67%) collected from 2001 to 2010 were grouped into cluster V_1_, which included nine distinct subclusters (V_1-1_–V_1-9_) when the similarity coefficient was 0.764. While the other 17 isolates, clustered into V_2_–V_13_, were quite different compared with other isolates and scattered in different years. These clusters contained rare isolates, such as SHRT_07-24_ (SHRT_07-24_ represents that this race is the isolate No. 24 from 2007, the same below), KHNS_08-25_, DBGN_05-18_, DHGK_02-17_, PHDT_08-13_, or LBGL_04-16_. The subcluster V_1-2_ was the largest subcluster consisting of 110 isolates and included three sub-subclusters (V_1-2-1_–V_1-2-3_) at a similarity coefficient of 0.793. The isolates collected from 2001 to 2006 were mainly distributed in the subclusters V_1-1_, V_1-2-1_, V_1-3_, and V_1-7_, and the isolates collected from 2007 to 2010 were mainly distributed in V_1-2-2_ and V_1-2-3_. These results indicated that the populations of *P. triticina* in Hebei Province from 2001 to 2010 had high polymorphism in virulence phenotypes. In addition, the virulence polymorphism had a certain correlation with the year, and the populations from 2007 to 2010 differed from those from 2001 to 2006.

**Figure 2:**
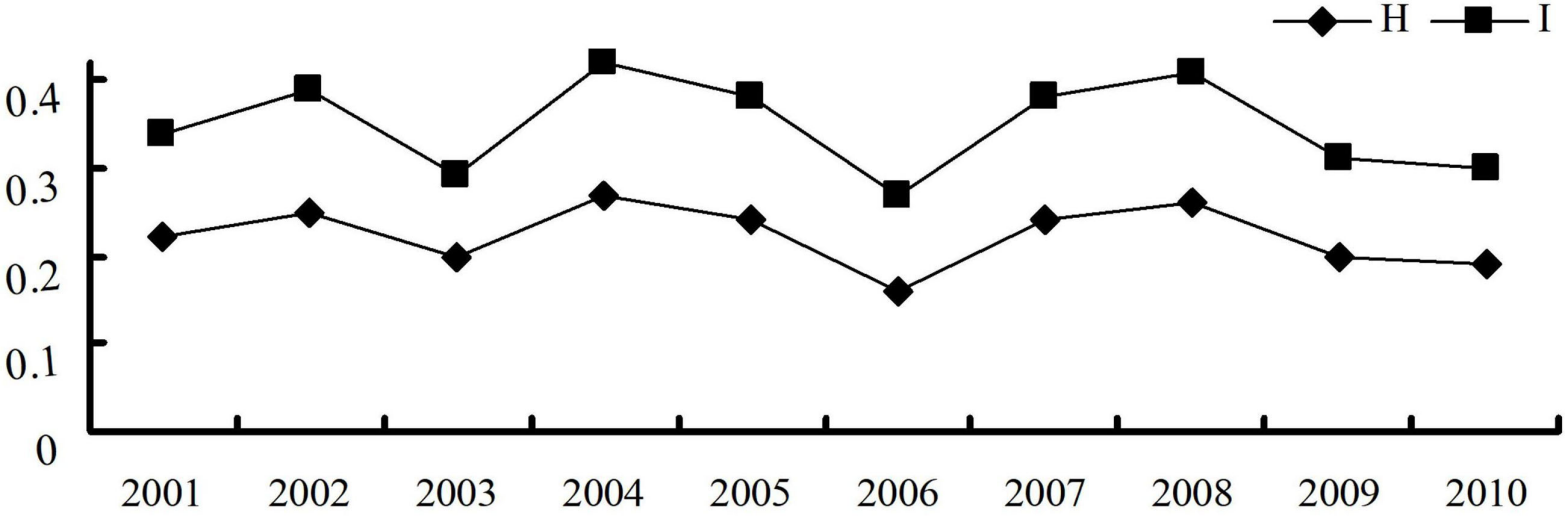
Virulence diversity of the *Puccinia triticina* populations in Hebei Province from 2001 to 2010 indicated by Nei’s gene diversity index (*H*) and Shannon’s information index (*I*).

The virulence diversity parameters Nei’s gene diversity index (*H*) and Shannon’s information index (*I*) of *P. triticina* populations from Hebei in 2001–2010 were 0.16–0.27 and 0.27–0.42, respectively. In addition, *H* and *I* had similar trends from 2001 to 2010 (**Table 2**), and both were in a state of fluctuation balance (**Figure 2**). This indicated that the virulence diversity of *P. triticina* populations in Hebei Province was relatively stable from 2001 to 2010.

**Table 2:** Virulence diversity parameters of 10 *Puccinia triticina* populations.

Parameters	2001	2002	2003	2004	2005	2006	2007	2008	2009	2010
Nei’s gene diversity index (*H*)	0.22	0.25	0.2	0.27	0.24	0.16	0.24	0.26	0.2	0.19
Shannon’s information index (*I*)	0.34	0.39	0.29	0.42	0.38	0.27	0.38	0.41	0.31	0.3

### Genetic polymorphism analysis

Two hundred forty-seven isolates of *P. triticina* were analyzed by 21 pairs of EST-SSR primers, and only 17 pairs of primers can amplify clear and stable bands (**Supplementary Figure S2**). In addition, three of the 17 pairs of primers, PtSSR0536, PtSSR3145, and PtSSR0801, did not amplify the polymorphism bands in the tested isolates. A total of 54 alleles were amplified from the remaining 14 pairs of primers, of which 42 alleles were polymorphism alleles, and the percentage of polymorphism was 77.78%. The number of alleles and polymorphic alleles of these 14 pairs of primers was 2–9 and 1–8, respectively (**Table 3**).

**Table 3:** Numbers of alleles, polymorphic alleles, and percentage of polymorphism of 14 pairs of EST-SSR primers.

Primer name	Number of alleles	Number of polymorphic alleles	Percentage of polymorphism (%)	Primer name	Number of alleles	Number of polymorphic alleles	Percentage of polymorphism (%)
PtSSR0083	2	1	50.00	PtSSR0189	3	2	66.67
PtSSR0019	2	1	50.00	PtSSR0481	3	2	66.67
PtSSR5649	5	4	80.00	PtSSR0639	4	3	75.00
PtSSR0085	3	2	66.67	PtSSR6542	9	8	88.89
PtSSR2948	4	3	75.00	PtSSR0182	3	2	66.67
PtSSR0243	3	3	100.00	PtSSR6386	7	6	85.71
PtSSR0125	2	1	50.00	average	3.86	3	77.78
PtSSR5594	4	4	100.00	Total	54	42	77.78

The genetic similarity coefficient based on EST-SSR analysis of the 247 isolates was 0.67–0.99 (**Supplementary Figure S3**), which was slightly higher than the virulence similarity coefficient. All isolates were clustered into four groups (S_1_–S_4_) at a similarity coefficient of 0.74. Cluster S1 contained 214 isolates (86.64%), including two distinct subclusters (S_1-1_ and S_1-2_) at a similarity coefficient of 0.758. The isolates of subcluster S_1-1_ and sub-subcluster S_1-2-1_ mainly came from 2001 to 2006, those of S_1-2-2_ were from 2009, those of S_1-2-3_ were from 2007, those of S_1-2-4_ and S_1-2-6_ were from 2008, and those of S_1-2-5_ were from 2010. While the isolates of clusters S_2_–S_4_ were from 2007 to 2009. These results indicated that the molecular genetic structure of *P. triticina* in Hebei Province from 2001 to 2006 was relatively consistent, and the genetic structure of *P. triticina* after 2007 had significantly changed compared with 2001–2006. Most of the isolates originating from the same or neighboring years were clustered into the same group or several neighboring subclusters with a high-level similarity, indicating that the genetic structure in these isolates might be related to their occurrence year.

### Two-dimensional principal coordinates analysis of EST-SSR and virulence

**Figure 3:**
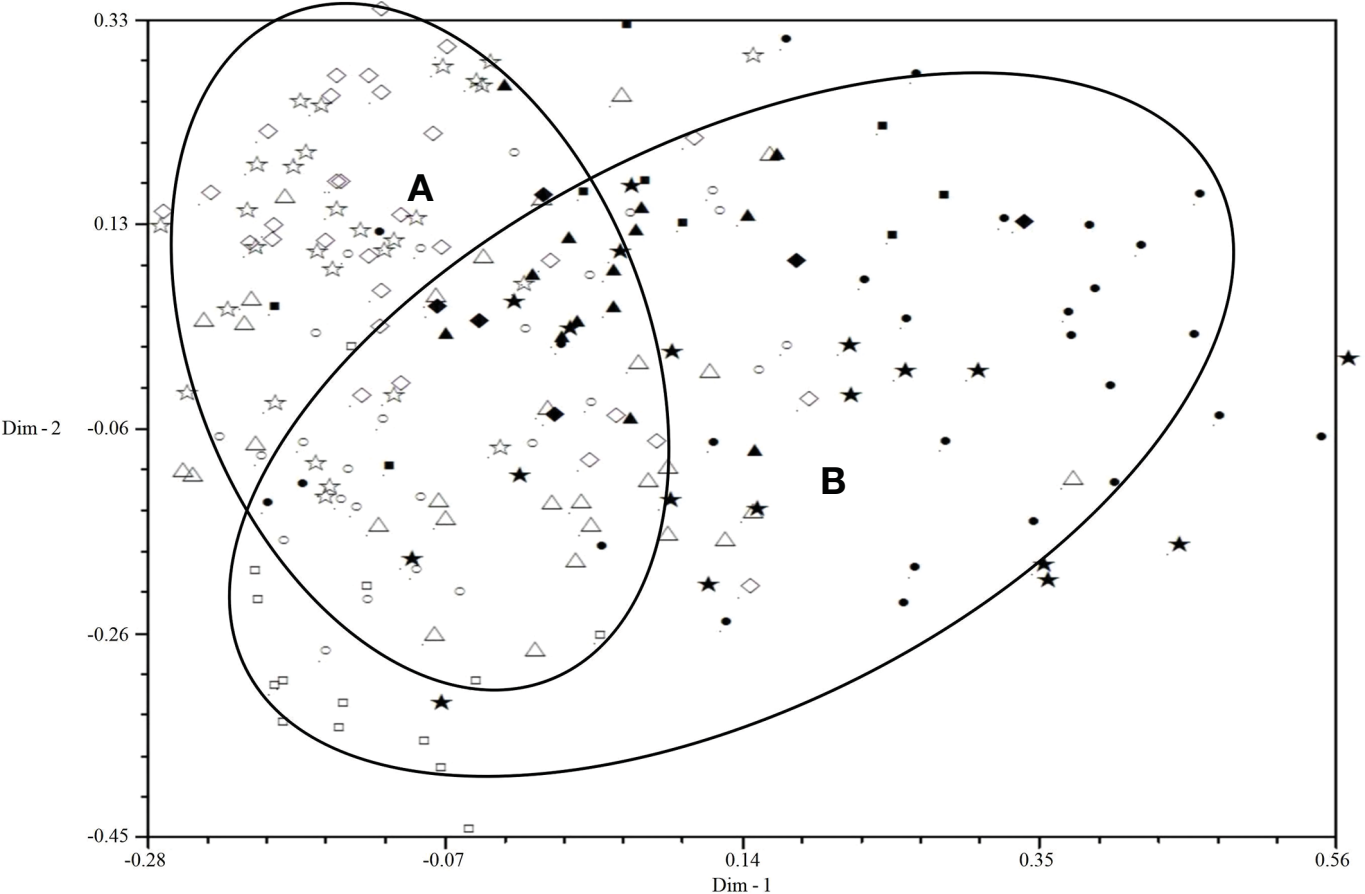
Two-dimensional plot of principal coordinates analysis of *Puccinia triticina* based on virulence polymorphism. ◼, Year 2001; ▲, Year 2002; ◆, Year 2003; •, Year 2004; ★, Year 2005; □, Year 2006; ○, Year 2007; △, Year 2008; ◇, Year 2009; ☆, Year 2010. Area A contains most isolates from 2007-2010 and area B contains most isolates from 2001-2006.

**Figure 4:**
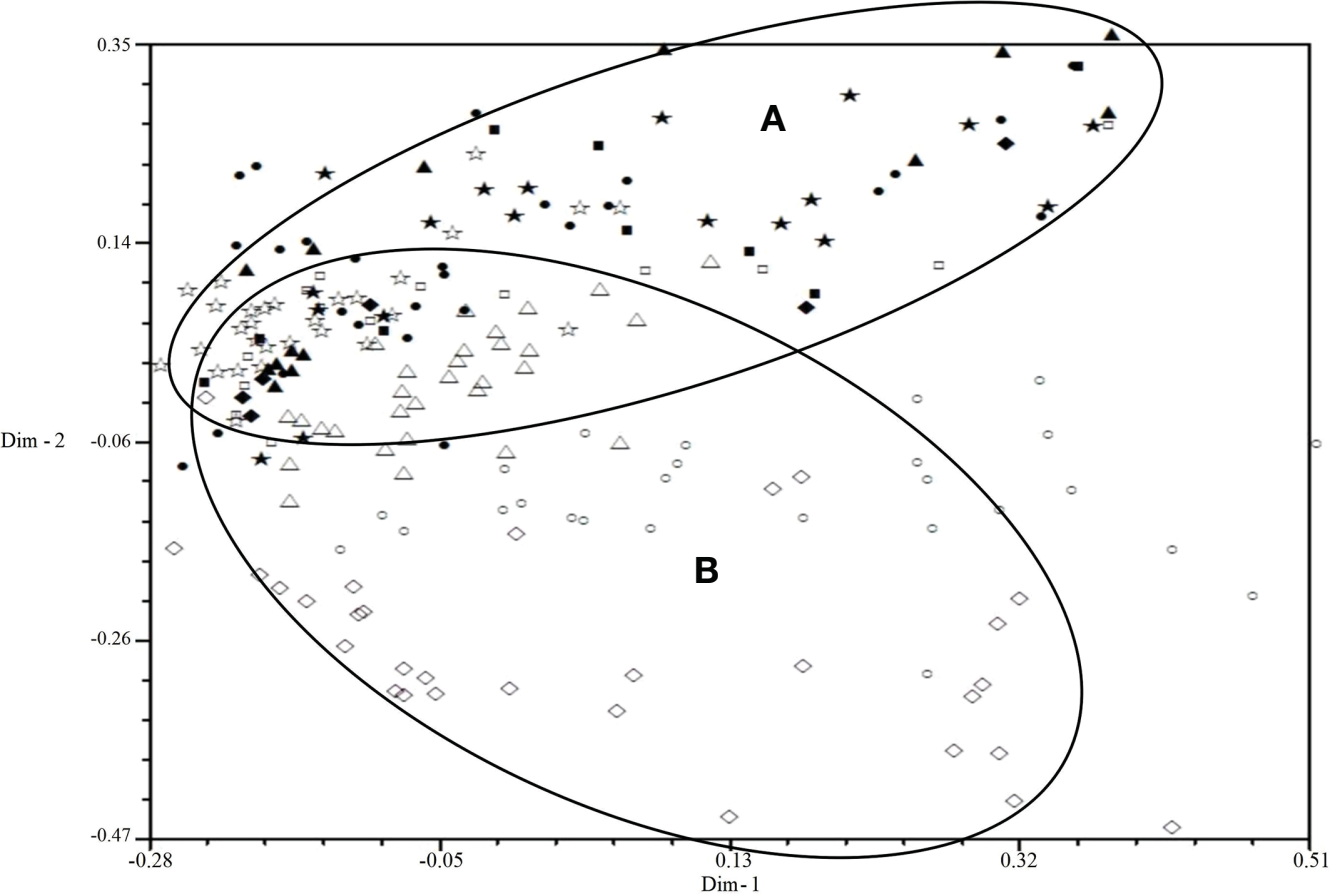
Two-dimensional plot of principal coordinates analysis of *Puccinia triticina* based on EST-SSR polymorphism. ◼, Year 2001; ▲, Year 2002; ◆, Year 2003; •, Year 2004; ★, Year 2005; □, Year 2006; ○, Year 2007; △, Year 2008; ◇, Year 2009; ☆, Year 2010. Area A contains most isolates from 2001-2006 and area B contains most isolates from 2007-2010.

All isolates were mainly distributed in two areas (A and B) by 2D-PCA plot analysis of virulence (**Figure 3**): the isolates from 2001 to 2006 were mainly distributed in area B, and the isolates from 2007 to 2010 were mainly distributed in area A, which also indicated that the population structure of *P. triticina* has changed after 2007 at a relatively macro level. In addition, the correlation coefficient of virulence and EST-SSR polymorphisms was only 0.006 by using MXCOMP module of NTSYS-pc, which indicated that the correlation between virulence and EST-SSR polymorphisms was very low. In the 2D-PCA plot analysis of EST-SSR, all isolates were mainly distributed in two areas (A and B) (**Figure 4**). The isolates from 2001 to 2006 were mainly concentrated in area A, which indicated that these isolates have similar genetic loci. While the isolates in 2007–2010 were mainly scattered and distributed in area B, which may suggest that the genetic diversity of *P. triticina* populations in 2007–2010 was relatively rich. Moreover, there was a partial overlap between area A and area B, which indicated that some isolates in *P. triticina* populations had a similar genetic structure in some years. The isolates of *P. triticina* in 2007–2010 were significantly different from those of other years, which may be due to the large changes in the genetic structure of *P. triticina* populations in these years.

### Virulence and EST-SSR polymorphism analysis of predominant races

**Figure 5:**
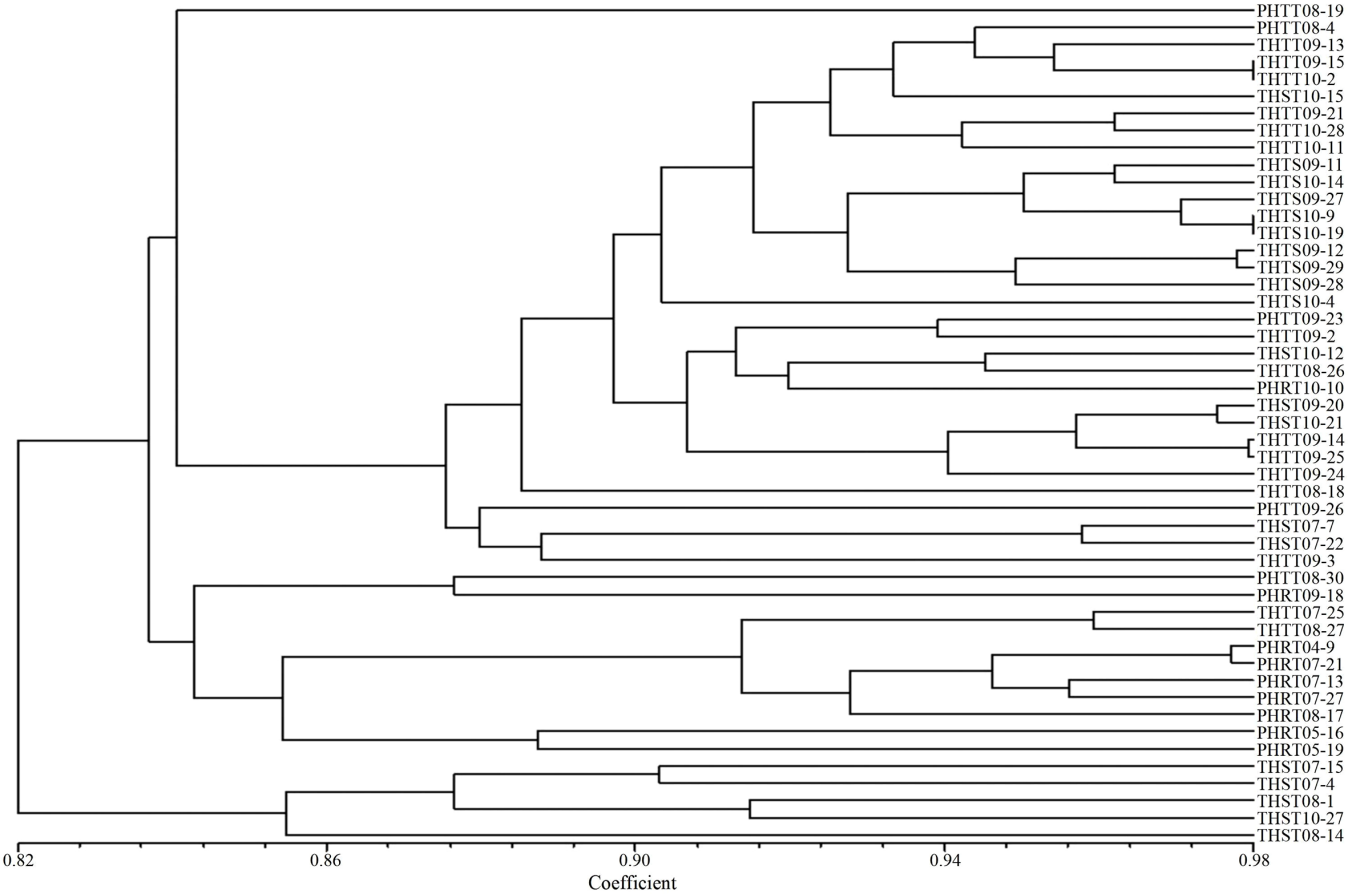
UPGMA dendrogram of the five *Puccinia triticina* predominant races (THTT, THST, THTS, PHRT, and PHTT) based on virulence.

**Figure 6:**
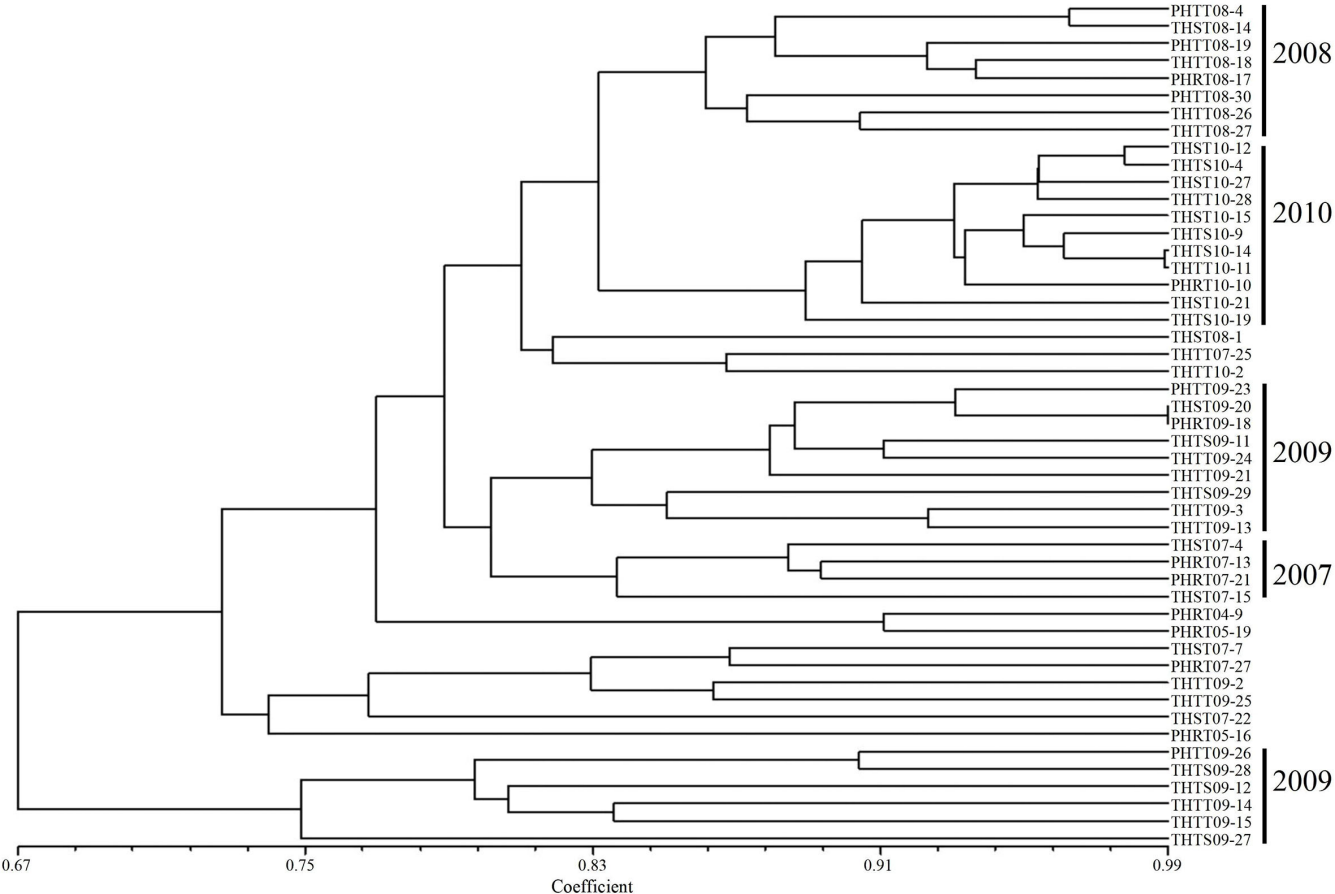
UPGMA dendrogram of the five *Puccinia triticina* predominant races (THTT, THST, THTS, PHRT, and PHTT) based on EST**-**SSR.

In order to further determine the relations of *P. triticina* pathotypes with virulence polymorphism or EST-SSR polymorphism, the cluster analysis was carried out on 49 isolates of five predominant races (THTT, THST, THTS, PHRT, and PHTT). In the polymorphism analysis based on virulence phenotypes, most of the same races tended to group in the same or adjacent clade (**Figure 5**). While in the polymorphism analysis based on EST-SSR, the races were clustered in different branches by year (**Figure 6**), which indicated that there was a certain genetic differentiation among or within the *P. triticina* population in different years. In addition, the correlation coefficient between pathotypes and virulence polymorphisms was 0.574 (>0.3), and the correlation coefficient between pathotypes and EST-SSR polymorphisms was 0.025 (<0.3), which was analyzed by the MXCOMP module of NTSYS-pc. These results indicated a correlation between pathotypes and virulence polymorphism, but there was no correlation between pathotypes and EST-SSR polymorphism.

### Genetic diversity analysis of *P. triticina* populations

The *P. triticina* isolates tested from 10 years (2001–2010) in Hebei Province have been designated as 10 corresponding populations by the year. The *H* of these *P. triticina* populations ranged from 0.13 to 0.26, and *I* ranged from 0.20 to 0.38, while the highest and lowest levels of genetic diversity were found in 2009 and 2010, respectively (**Table 4**). From the trend of these results, the population genetic diversity level was relatively stable in 2001–2006, and the genetic diversity of the population showed obvious fluctuations in 2007–2010.

**Table 4:** Genetic diversity parameters of 10 *Puccinia triticina* populations based on EST-SSR.

Populations	Genetic polymorphic parameters					
	Observed number of alleles (*N_a_*)	Effective number of alleles (*N_e_*)	Nei’s gene diversity index (*H*)	Shannon’s information index (*I*)	Number of polymorphic loci (*NP*)	Percentage of polymorphic loci (*P* %)
2001	1.58	1.34	0.20	0.30	35	58.33
2002	1.55	1.31	0.19	0.28	33	55.00
2003	1.40	1.28	0.16	0.23	24	40.00
2004	1.65	1.34	0.20	0.31	39	65.00
2005	1.58	1.32	0.19	0.29	35	58.33
2006	1.55	1.31	0.19	0.28	33	55.00
2007	1.65	1.43	0.25	0.36	39	65.00
2008	1.48	1.29	0.17	0.25	29	48.33
2009	1.73	1.46	0.26	0.38	44	73.33
2010	1.43	1.21	0.13	0.20	26	43.33
Species level	1.87	1.46	0.27	0.41	52	86.67
Population level	1.56	1.33	0.19	0.29	33.7	56.17

In addition, the analysis results of POPGENE software showed that the coefficient of genetic differentiation (Gst) was 0.27, of which genetic diversity within the population accounts for 73.08% of the total genetic diversity, and genetic diversity among the populations accounts for 26.92% of the total population genetic diversity. There were also certain genetic differentiations among or within *P. triticina* populations analyzed by the Arlequin3.ll software, and the genetic variation within populations accounted for 72.54% of the total variation, and the genetic variation among the populations accounted for 27.46% (**Table 5**). So, the variations were mainly found within populations of *P. triticina*. Moreover, a certain level of gene flow (*N_m_* = 1.38 > 1) was detected by POPGENE software among the populations in 2001–2010. These results indicated that there was a certain degree of exchange of inoculum sources among populations of different years, which may play a role in the changes of population genetic structures.

**Table 5:** Analysis of molecular variance (AMOVA) among and within populations.

Source of variation	Degree of freedom	Sum of squares	Percentage of variation	*P* value
Among populations	9	566.19	27.46	<0.0001
Within populations	237	1,452.36	72.54	<0.0001
Total	246	2,018.55	100.00	

**Figure 7:**
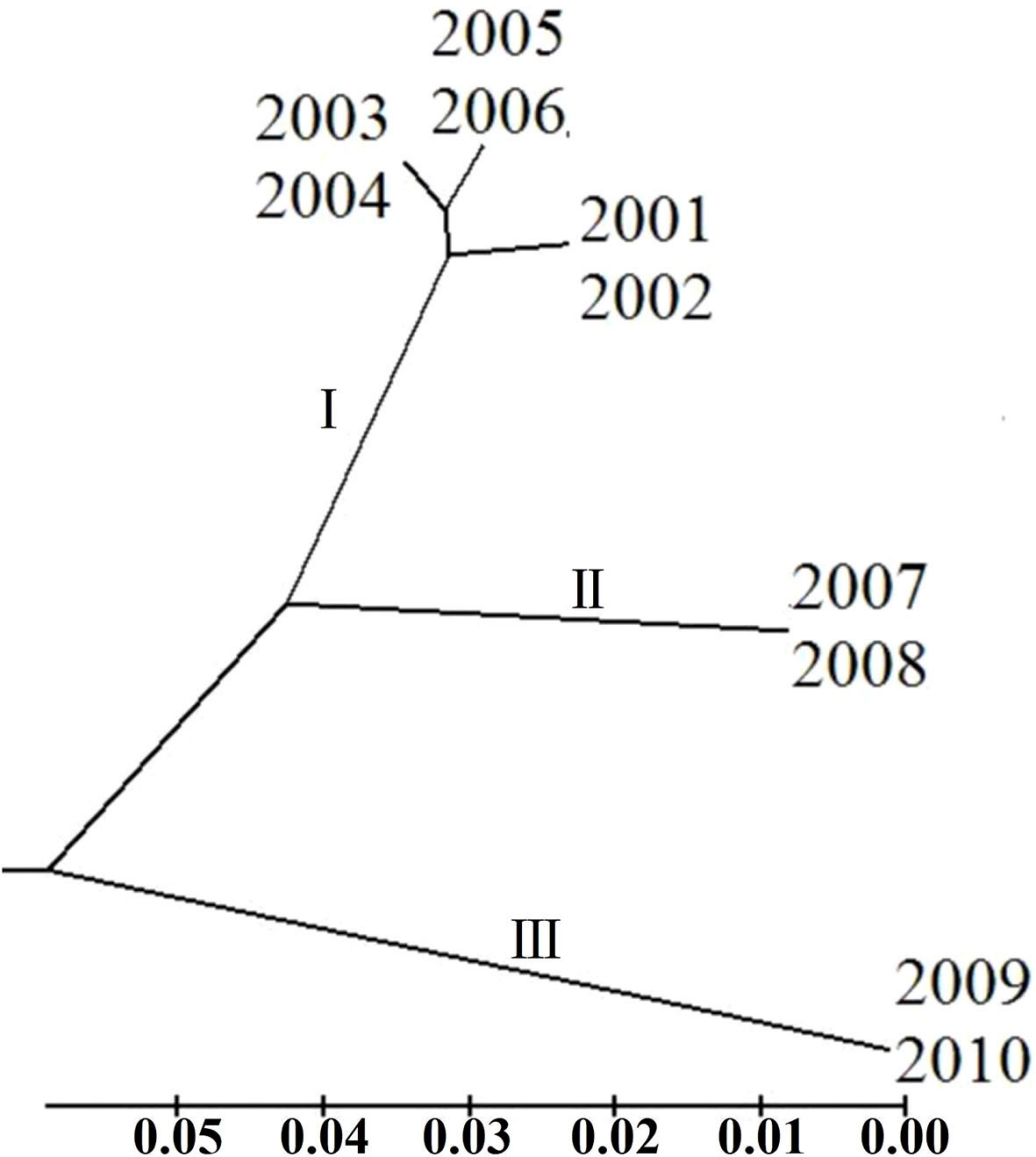
Phylogenetic tree of the 10 *Puccinia triticina* populations based on EST-SSR. The isolates of each year are regarded as a population.

To clarify the genetic relationship between different *P. triticina* populations from 2001 to 2010, the population genetic distance was estimated using the POPGENE software (**Table 6**). A dendrogram (UPGMA, unweighted average method) was then constructed by MEGA 6.0 software based on the Nei’s genetic distances (**Figure 7**). The 10 populations of *P. triticina* could be clustered into three main branches (I–III), 2001–2006, 2007–2008, and 2009–2010. The genetic relationship of the six populations from 2001 to 2006 was relatively closer, while the two populations from 2009 and 2010 had the farthest relationship with other populations, followed by the two populations in 2007–2008. This indicated that the longer the year span, the greater the genetic difference of *P. triticina* populations.

**Table 6:** Nei’s unbiased measures of genetic distances and genetic identities between populations.

Populations	2001	2002	2003	2004	2005	2006	2007	2008	2009	2010
2001	****	0.9772	0.9643	0.9749	0.9831	0.9569	0.9026	0.8927	0.8453	0.8774
2002	0.0231	****	0.9926	0.9872	0.9930	0.9797	0.8931	0.9125	0.8554	0.8657
2003	0.0363	0.0074	****	0.9862	0.9874	0.9828	0.8911	0.9181	0.8907	0.8414
2004	0.0255	0.0129	0.0139	****	0.9866	0.9723	0.8744	0.8988	0.8565	0.8555
2005	0.0171	0.0070	0.0126	0.0135	****	0.9750	0.9028	0.8995	0.8525	0.8611
2006	0.0440	0.0206	0.0174	0.0280	0.0253	****	0.8847	0.8928	0.8766	0.8476
2007	0.1025	0.1131	0.1153	0.1342	0.1023	0.1225	****	0.8611	0.9147	0.8258
2008	0.1135	0.0916	0.0855	0.1067	0.1059	0.1134	0.1496	****	0.8670	0.8852
2009	0.1681	0.1562	0.1158	0.1549	0.1596	0.1318	0.0892	0.1427	****	0.7925
2010	0.1308	0.1442	0.1727	0.1561	0.1495	0.1654	0.1914	0.1220	0.2326	****

Nei’s genetic identities (above diagonal) and genetic distances (below diagonal).

## Discussion

The most common method to study population dynamics of *P. triticina* is to monitor the virulence changes based on the infection types of isolates to a set of Thatcher near-isogenic lines or currently planted cultivars, which can directly reflect the changes of pathotypes and virulence of *P. triticina*. While molecular marker technology can reveal the genetic structure, genetic differentiation, and genetic diversity of pathogens at the molecular level, which can reflect the difference between individuals of pathogens based on the variation of gene or genotype (Park et al., 2000; Xu et al., 2002; Mebrate et al., 2006; Kolmer and Ordoñez, 2007; Mantovani et al., 2010; Ordoñez et al., 2010). In addition, molecular marker technology is also widely used in the virulence variation, origin evolution, and migration of pathogens (Chen et al., 1999; Xu et al., 2009). In this study, the genetic diversity, evolution, and variation of *P. triticina* from 2001 to 2010 in Hebei Province were researched by combining virulence identification with EST-SSR analysis.

A relatively rich genetic diversity was found among the *P. triticina* isolates from 2001 to 2010. However, the genetic structure of *P. triticina* populations varied with the year difference. The population structure of *P. triticina* underwent major changes in 2007–2010, while the populations of 2001–2006 were similar and had little change (**Figure 7**). In general, the factors affecting the genetic structure of *P. triticina* populations are mainly due to the mutation of the pathogen, the alternation of host cultivars, and the climate environment changes. According to our surveys during the collection period of leaf rust samples from 2001 to 2010, the wheat-planting area in Hebei Province decreased since 2001 due to various reasons. The sown area of wheat in Hebei Province was 2,729.9 thousand hectares in 2000, decreased to 2,161.5 thousand hectares in 2003 and 2,377.1 thousand hectares in 2004, and farmers rarely changed wheat cultivars during these years. The wheat cultivars of ‘Shimai’ series, ‘Hengmai’ series, and ‘Hanmai’ series accounted for more than 80% of the wheat-planting area in Hebei Province (Chen et al., 2005; Zhang, 2016). For example, ‘Shixin733,’ ‘Shijiazhuang 8,’ ‘Shimai 12,’ ‘Shimai 16,’ ‘Shixin 828,’ ‘Shimai 14,’ ‘Hengguan 35,’ ‘Hanmai 11,’ Hanmai 12,’ and ‘Ji 5265’ were the main wheat cultivars during this period, and these cultivars were all native wheat cultivars in Hebei Province. Since 2005, the wheat-planting area in Hebei Province has increased to 2,420 thousand hectares, which has remained at this level in the following years, and some new wheat cultivars were introduced. For example, ‘Jimai 22’ and ‘Liangxing 99,’ which belong to the ‘Jimai’ series and ‘Liangxing’ series from Shandong Province, Zhongmai 175, and so on were introduced into Hebei Province and have a wide planting area for high yield characterization. The alternation of wheat cultivars in this periods may produce affections on *P. triticina* population structure changes. In addition, the precipitations, temperatures, and altitude can also affect the development of *P. triticina* populations (Morgounov et al., 2012). According to the Climate Bulletin of Hebei Province, the annual average temperature in Hebei Province was 0.6°C–1.3°C higher than normal in 2007–2010, which were the warmest years since meteorological observations, and the winter temperature was significantly higher, which was the second warmest winter since 1951. Warm winter will be beneficial to the overwintering and survival rate of *P. triticina*, which may be conducive to the emergence of new races and the development of the population size of *P. triticina*. Since the period of large changes in the population structure of *P. triticina* was consistent with the changes in climate conditions, the warm winter may be another reason for the changes in the population structure of *P. triticina* in 2007–2010. Based on the above analysis, we speculated that the changes in *P. triticina* population structure during 2001–2010 were caused by the combined effects of host cultivars and climate conditions.

Kosman et al. (Kosman et al., 2014; Kosman et al., 2019) and Kolmer et al. (2017) thought that migration was also likely an important factor in the genetic structure change of *P. triticina* populations. Wright (1990) proposed that when parameter *N_m_* (gene flow) >1, there was gene flow between populations, which could prevent genetic differentiations between populations. Gene flow has the role of homogenization, and the stronger the gene flow, the higher the similarity and the smaller the differentiation. On the contrary, the lower the gene flow, the lower the similarity and the larger the differentiation. The gene flow generated by the spread and diffusion of spores in different regions may change the gene frequency of the population. In addition, the interannual gene flow was mainly caused by asexual spores overwintering and oversummering, which was generally relatively stable, but low temperature or drastic environmental changes may also cause variations. The north and northwest of Hebei Province are located in mountainous areas, which have fewer wheat-planting areas, so no samples were collected for identification. The main wheat-growing region of Hebei Province is in the northern part of the North China Plain (only including Hebei Province), which is narrow compared with the southern part of the North China Plain (including Henan Province, Shandong Province, Anhui Province, and North part of Jiangsu Province). So, the plain is very conducive to the migration of *P. triticina* by the monsoon, and the gene flow caused by the migration of *P. triticina* among regions may reduce the diversity of *P. triticina* in the same year. Therefore, the difference of *P. triticina* population in the same year may come from the difference of wheat cultivars planted in different regions.

Wang et al. (Wang et al., 2010a, Wang et al. 2010b) developed 21 pairs of EST-SSR primers, which were effective in analyzing the molecular diversity of *P. triticina* isolates in Canada. However, similar to the study of Zhao (2013), four pairs of primers in the present study cannot amplify clear and stable bands. In addition, although three pairs of primers can amplify clear and stable bands, there are no polymorphic bands, which cannot distinguish the isolates of *P. triticina*. So, only 14 pairs of EST-SSR primers in this study were adapted to the genetic diversity analysis of *P. triticina* in China. Due to the significant difference in virulence of *P. triticina* between North America and China, we speculated that the genetic difference of *P. triticina* between the two regions may be the main reason for this phenomenon.

Many studies on the genetic diversity of *P. triticina* populations showed that the genetic variations were mainly found within populations, which was higher than among populations (Kolmer and Ordoñez, 2007; Ordoñez and Kolmer, 2007; Ordoñez et al., 2010; Dadrezaie et al., 2013; Xu et al., 2013). Moreover, similar results were reported in the *Puccinia striiformis* f. sp. *tritici* (Wan et al., 2015). In this study, we found a certain degree of genetic differentiation among populations or within populations. The variation within populations was higher than that among populations of *P. triticina* in Hebei Province from 2001 to 2010. These results were consistent with the above studies. This indicated that the differentiation within populations in the same year played a more important role than the differentiation among populations, revealing that year separation was also apparently an important limiting factor to gene flow and population structure. Because the overwintering survival rate of *P. triticina* in Hebei Province is relatively low, the source of the *P. triticina* inoculum in Hebei Province, particularly north of Shijiazhuang, may have migrated primarily from other major wheat-growing regions that are conducive to *P. triticina* overwintering, such as Henan, Shandong, and Jiangsu Province, which are located in the middle-south of the North China Plain. The main wheat cultivars in these provinces are different. For example, the wheat cultivars of Henan Province are mainly ‘Zhengmai’ series and ‘Zhoumai’ series, the wheat cultivars of Shandong Province are mainly ‘Jimai’ series, ‘Lumai’ series, and ‘Yanmai’ series, and the wheat cultivars of Jiangsu Province are mainly ‘Sumai’ series and ‘Yangmai’ series, which also easily lead to the diversity of *P. triticina* races. Therefore, the genetic variation of *P. triticina* in Hebei Province from 2001 to 2010 was mainly found within populations, which may be caused by the differences of races from other regions outside Hebei Province mentioned above. In addition, according to the study of Dadrezaie et al. (2013), although the high molecular variability within *P. triticina* populations may indicate the colonial behavior of *P. triticina* in different years, the similarity between *P. triticina* populations also indicates the gene flow. So the population similarity between years may be the result of gene flow.

According to the virulence and genetic polymorphism analysis, we found that the diversity of *P. triticina* in Hebei Province was rich, and the virulence structure was very complex. Both virulence and genetic polymorphism analyses showed that the clustering of some *P. triticina* isolates in Hebei Province had a certain correlation with the year, which indicated that the genotypes of the *P. triticina* isolates in the same year had more similarity than that in different years. Both analyses showed significant changes in the population structure of *P. triticina* in Hebei Province after 2007 compared with 2001–2006. With the change in climate, the occurrence of wheat leaf rust in China has been increasing yearly, especially after 2008, such as 2008–2009, 2012–2013, and 2015 (Zhang et al., 2020a; Zhang et al., 2020b). The increase in *P. triticina* population size will increase the possibility of survival of individual isolates with favorable mutations for virulence or at genetic loci (Kolmer, 2015), which will further enrich the population diversity of *P. triticina*. In addition, we found that there was no significant correlation between these isolates and the regions, which further indicated the migration of *P. triticina* isolates among these regions.

Moreover, there is a contradiction in the correlation between virulence phenotypes and molecular genotypes of *P. triticina* in the world. Many studies on genetic diversity have reported the significant correlation between the virulence phenotypes and molecular genotypes of *P. triticina* in Pakistan, Europe, Central Asia, and the Caucasus regions (Halkett et al., 2005; Kolmer and Ordoñez, 2007; Mantovani et al., 2010; Kolmer et al., 2012; Kolmer et al., 2017). However, Park et al. (2000); Prasad et al. (2017), and Gultyaeva et al. (2018) thought that there was no significant correlation between virulence polymorphism and molecular polymorphism of the *P. triticina* population diversity in Western Europe, South Asia, or Russia and Kazakhstan. There was also no significant correlation between virulence polymorphism and molecular polymorphism of the *P. triticina* population in China (Xu et al., 2002; Pu et al., 2004; Xu et al., 2013; Zhao, 2013), which was consistent with the results of this study. In addition, the same conclusion had been reported in the studies of population genetics of *P. striiformis* f. sp. *tritici* in the United States and Northwestern China (Chen et al., 2010; Wan et al., 2015). Whereas Kolmer (2015) reported that the SSR genotypes of 100 P*. triticina* isolates from seven provinces (the samples provided by our lab) in 2009 and 2010 in China had a significant correlation with the virulence phenotypes, which may be related to the sample size or the regional source differences of the collections of *P. triticina*. The greater regional disparity, the greater possibility of virulence and genetic variation of *P. triticina* populations.

In this study, the results of polymorphism analysis of the predominant pathotypes showed a correlation between pathotype and virulence polymorphism. However, the different pathotypes may also be grouped into the same cluster. For example, THTT and PHRT or PHTT and PHRT, which differed in virulence on *Lr2a* and/or *Lr17*, were clustered into the same cluster. Similar phenomena have been found in other studies. Wang et al. (2010a) found that TDBG and TDBJ, which differed in virulence on *Lr14a*, were clustered into the same cluster. While some of the same races did not completely cluster into the same group, which indicated that these isolates have genetic differences, although they were of the same pathotype. Zhao and Wang (Zhao and Wang, 1986; Wang, 1988) found the virulence heterogeneity in the same pathotype of *P. triticina*, and the pathogenicity of different *P. triticina* isolates of the same pathotype was not completely consistent. The more differential hosts were used, the more pathotypes of *P. triticina* were identified. In this study, in addition to 16 standardized differentials that were used for race naming, 20 extra differentials were used to test the virulence of *P. triticina* isolates. Therefore, although the same races had the same infection types to those of 16 standard hosts, there may be differences in the infection types to additional extra differentials. For example, isolate THTT_10-2_ was virulent to *Lr15* and avirulent to *Lr28* and *Lr36*, but THTT_09-25_ was avirulent to *Lr15* and virulent to *Lr28* and *Lr36*. THTT_09-2_ was avirulent to *Lr28* and *Lr33+34* and virulent to *Lr15* and *Lr36*, but THTT_08-27_ was virulent to *Lr28* and *Lr33+34* and avirulent to *Lr15* and *Lr36*. Therefore, although these isolates are of the same pathotype according to the 4-letter nomenclature, some of them were different in the virulence to other *Lr* genes. With the increase of the differential hosts for pathotype identification, the diversity of isolates may be more abundant.

## Conclusion

In the present study, we identified the virulence diversity of 247 P*. triticina* isolates collected in Hebei Province from 2001 to 2010 on 36 Thatcher near-isogenic lines and detected the EST-SSR genetic diversity. The cluster analysis based on virulence phenotype and EST-SSR genotype showed that *P. triticina* populations in Hebei Province have rich virulence and genetic diversity. Due to the alternation of wheat cultivars and climatic condition changes, the population structure of *P. triticina* has changed significantly since 2007. Moreover, there was a certain genetic differentiation among or within populations of *P. triticina* in Hebei Province, and the genetic variation of within populations was the main origin of virulence phenotype or genetic genotype variations in *P. triticina* populations in Hebei Province. Our study will provide a theoretical basis for a better understanding of the population structure and genetic variations of *P. triticina* over a period in Hebei Province of China.

## Data availability statement

The original contributions presented in the study are included in the article/**Supplementary Material**. Further inquiries can be directed to the corresponding authors.

## Author contributions

HY and DL conceived and designed the research. LZ and LYZ performed the experiments and analyzed the data. QM participated and assisted in the virulence identification of isolates. LZ, LYZ, and HY wrote the manuscript. HY and DL reviewed and edited the manuscript. All authors contributed to the article and approved the submitted version.
