# Supplementary figures and images for: Virulence and molecular genetic diversity, variation, and evolution of the Puccinia triticina population in Hebei Province of China from 2001 to 2010
Source: Front Plant Sci. 2023 Mar 6;14:1095677. doi: 10.3389/fpls.2023.1095677 (PMC10025498; doi:10.3389/fpls.2023.1095677)

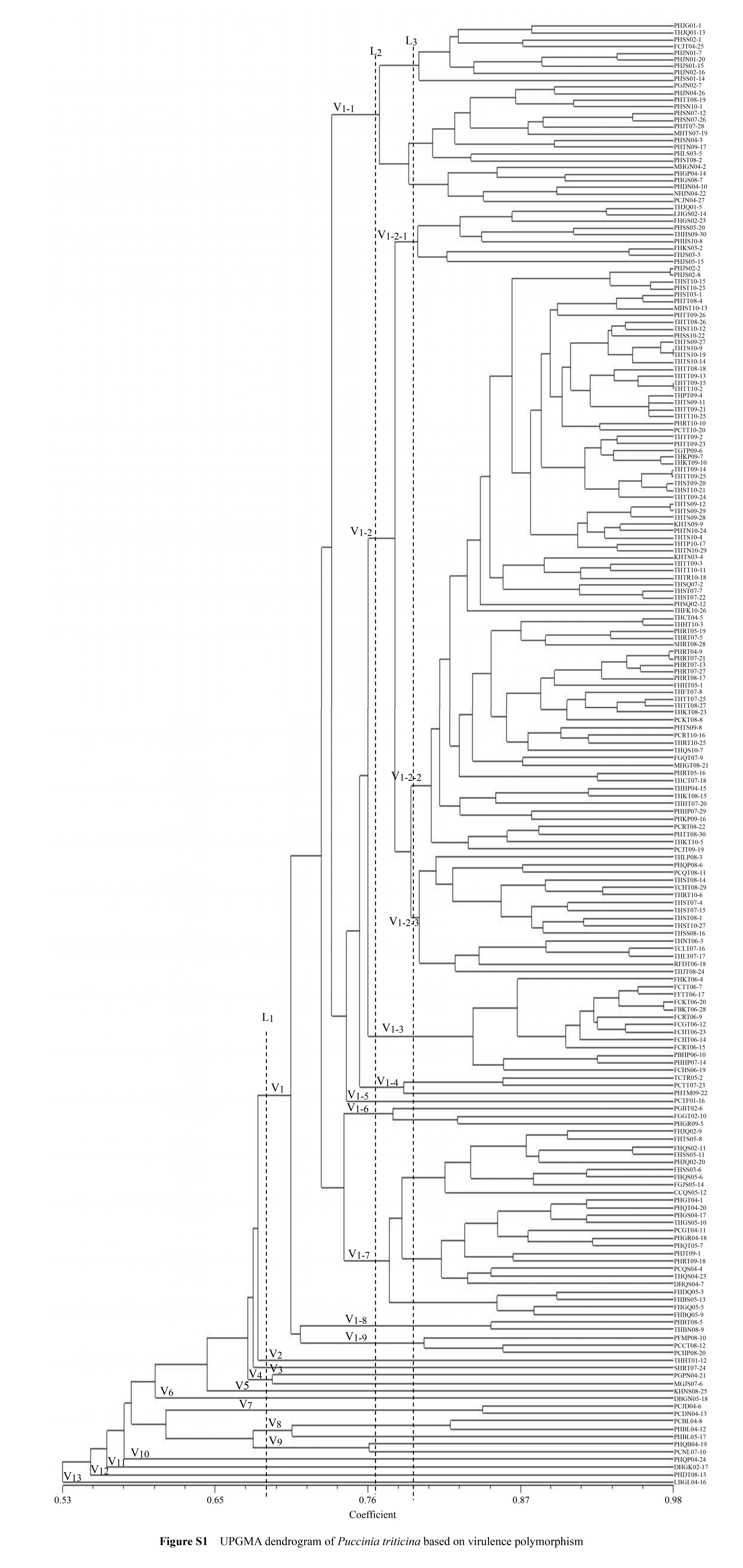

Supplement: Supplementary file 1 [file Image_1.jpeg]

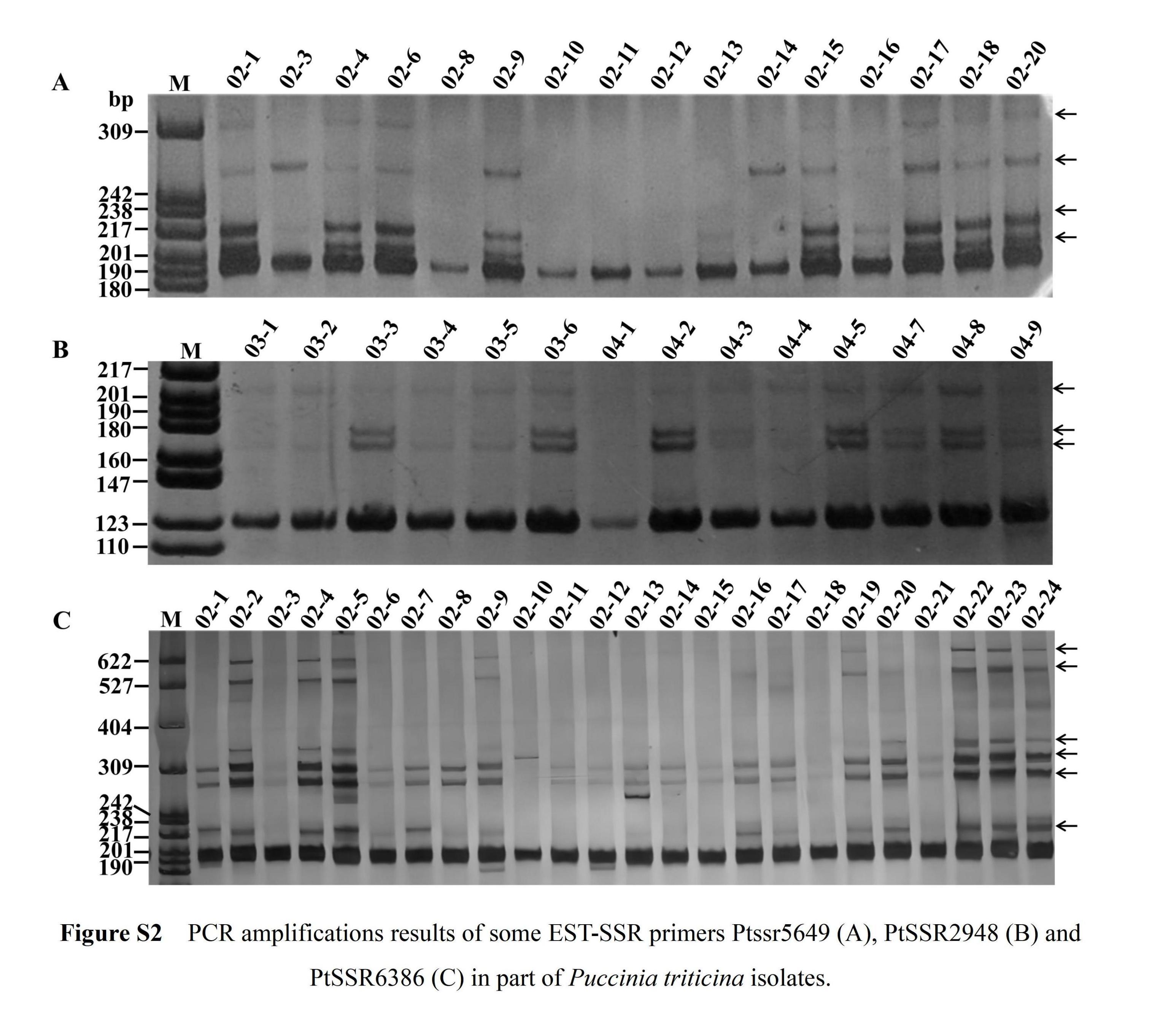

Supplement: Supplementary file 2 [file Image_2.jpeg]

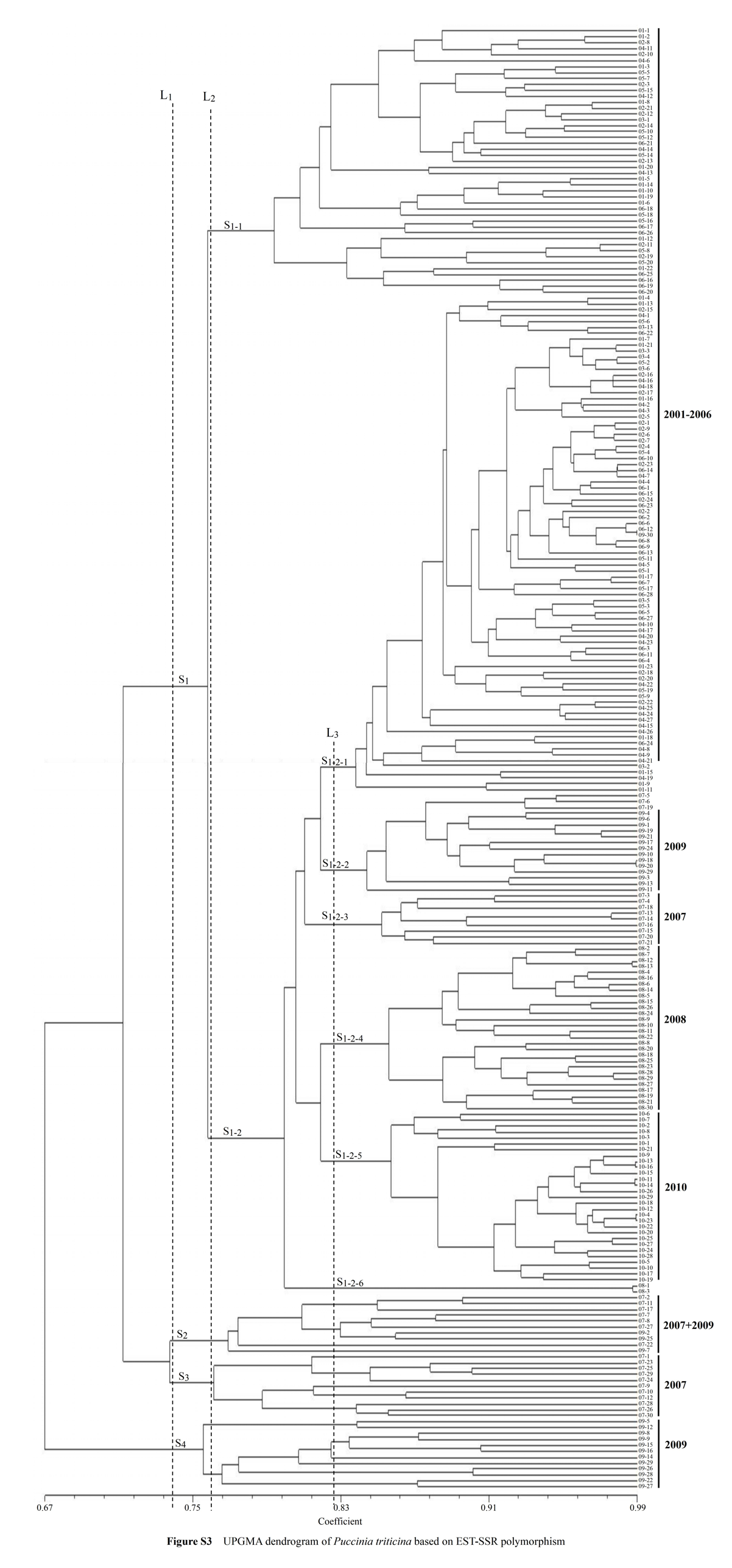

Supplement: Supplementary file 3 [file Image_3.jpeg]
