# Supplementary material for: Virulence and molecular genetic diversity, variation, and evolution of the Puccinia triticina population in Hebei Province of China from 2001 to 2010
Source: Front Plant Sci. 2023 Mar 6;14:1095677. doi: 10.3389/fpls.2023.1095677 (PMC10025498; doi:10.3389/fpls.2023.1095677)
Supplement: Supplementary file 4 [file Table_1.pdf]

TABLE S1 Isolate code, location and race information of *Puccinia triticina* samples collected from 10 regions of Hebei Province from 2001 to 2010.

Isolate Codes	Year	Region	Races	Isolate Codes	Year	Region	Races	Isolate Codes	Year	Region	Races
01-1	2001	Handan	PHJG	05-4	2005	Baoding	—	08-9	2008	Cangzhou	THBN
01-2	2001	Baoding	—	05-5	2005	Langfang	FHGQ	08-10	2008	Cangzhou	PFMP
01-3	2001	Langfang	—	05-6	2005	Baoding	FHQS	08-11	2008	Cangzhou	PCQT
01-4	2001	Baoding	—	05-7	2005	Shijiazhuang	PHQT	08-12	2008	Cangzhou	PCCT
01-5	2001	Handan	THJQ	05-8	2005	Baoding	FHTS	08-13	2008	Cangzhou	PHDT
01-6	2001	Baoding	—	05-9	2005	Baoding	FHBQ	08-14	2008	Cangzhou	THST
01-7	2001	Baoding	PHJN	05-10	2005	Langfang	THGS	08-15	2008	Cangzhou	THKT
01-8	2001	Shijiazhuang	—	05-11	2005	Baoding	FHSS	08-16	2008	Cangzhou	THSS
01-9	2001	Shijiazhuang	—	05-12	2005	Xingtai	CCQS	08-17	2008	Baoding	PHRT
01-10	2001	Shijiazhuang	—	05-13	2005	Baoding	FHBS	08-18	2008	Baoding	THTT
01-11	2001	Handan	—	05-14	2005	Baoding	FGJS	08-19	2008	Baoding	PHTT
01-12	2001	Cangzhou	THHT	05-15	2005	Xingtai	PHJS	08-20	2008	Baoding	PCHP
01-13	2001	Shijiazhuang	THJQ	05-16	2005	Baoding	PHRT	08-21	2008	Baoding	MHGT
01-14	2001	Baoding	PHSS	05-17	2005	Hengshui	PHBL	08-22	2008	Baoding	PCRT
01-15	2001	Baoding	PHJS	05-18	2005	Baoding	DBGN	08-23	2008	Baoding	THKT
01-16	2001	Baoding	PCTF	05-19	2005	Baoding	PHRT	08-24	2008	Baoding	THJT
01-17	2001	Baoding	—	05-20	2005	Shijiazhuang	PHSS	08-25	2008	Baoding	KHNS
01-18	2001	Baoding	—	06-1	2006	Shijiazhuang	—	08-26	2008	Shijiazhuang	THTT
01-19	2001	Baoding	—	06-2	2006	Hengshui	—	08-27	2008	Shijiazhuang	THTT
01-20	2001	Baoding	PHJN	06-3	2006	Hengshui	THNT	08-28	2008	Shijiazhuang	SHRT
01-21	2001	Baoding	—	06-4	2006	Hengshui	FHKT	08-29	2008	Shijiazhuang	TCHT
01-22	2001	Cangzhou	—	06-5	2006	Hengshui	—	08-30	2008	Shijiazhuang	PHTT
01-23	2001	Handan	—	06-6	2006	Hengshui	—	09-1	2009	Tangshan	PHJT
02-1	2002	Baoding	PHSS	06-7	2006	Hengshui	FCTT	09-2	2009	Tangshan	THTT
02-2	2002	Baoding	PHJS	06-8	2006	Hengshui	—	09-3	2009	Tangshan	THTT
02-3	2002	Baoding	—	06-9	2006	Hengshui	FCRT	09-4	2009	Langfang	THPT
02-4	2002	Baoding	—	06-10	2006	Hengshui	PBHP	09-5	2009	Baoding	PHGR
02-5	2002	Xingtai	—	06-11	2006	Handan	—	09-6	2009	Baoding	TGTP
02-6	2002	Baoding	PGBT	06-12	2006	Handan	FCGT	09-7	2009	Shijiazhuang	THKP
02-7	2002	Baoding	PGJN	06-13	2006	Handan	—	09-8	2009	Langfang	PHTS
02-8	2002	Baoding	PHJS	06-14	2006	Handan	FCHT	09-9	2009	Shijiazhuang	KHTS
02-9	2002	Baoding	FHJQ	06-15	2006	Hengshui	FCRT	09-10	2009	Handan	THKT
02-10	2002	Baoding	FGGT	06-16	2006	Baoding	—	09-11	2009	Tangshan	THTS
02-11	2002	Xingtai	FHQS	06-17	2006	Baoding	FFTT	09-12	2009	Handan	THTS
02-12	2002	Baoding	PHSQ	06-18	2006	Handan	RFDT	09-13	2009	Handan	THTT
02-13	2002	Baoding	—	06-19	2006	Cangzhou	FCHS	09-14	2009	Handan	THTT
02-14	2002	Baoding	LHGS	06-20	2006	Hengshui	FCKT	09-15	2009	Shijiazhuang	THTT
02-15	2002	Baoding	—	06-21	2006	Shijiazhuang	—	09-16	2009	Tangshan	PHKP
02-16	2002	Baoding	PHJN	06-22	2006	Shijiazhuang	—	09-17	2009	Handan	PHTN
02-17	2002	Baoding	DHGK	06-23	2006	Hengshui	FCHT	09-18	2009	Xingtai	PHRT
02-18	2002	Baoding	—	06-24	2006	Shijiazhuang	—	09-19	2009	Xingtai	PCJT

02-19	2002	Baoding	—	06-25	2006	Cangzhou	—	09-20	2009	Xingtai	THST
02-20	2002	Baoding	PHJQ	06-26	2006	Cangzhou	—	09-21	2009	Langfang	THTT
02-21	2002	Baoding	—	06-27	2006	Baoding	—	09-22	2009	Xingtai	PHTM
02-22	2002	Baoding	—	06-28	2006	Handan	FBKT	09-23	2009	Hengshui	PHTT
02-23	2002	Baoding	FHGS	07-1	2007	Baoding	—	09-24	2009	Hengshui	THTT
02-24	2002	Baoding	—	07-2	2007	Baoding	THSQ	09-25	2009	Handan	THTT
03-1	2003	Tangshan	PHST	07-3	2007	Baoding	—	09-26	2009	Tangshan	PHTT
03-2	2003	Cangzhou	FHKS	07-4	2007	Hengshui	THST	09-27	2009	Xingtai	THTS
03-3	2003	Langfang	FHJS	07-5	2007	Hengshui	THRT	09-28	2009	Hengshui	THTS
03-4	2003	Shijiazhuang	KHTS	07-6	2007	Hengshui	MGJS	09-29	2009	Hengshui	THTS
03-5	2003	Chengde	PHLS	07-7	2007	Hengshui	THST	09-30	2009	Cangzhou	THHS
03-6	2003	Baoding	FHSS	07-8	2007	Hengshui	THFT	10-1	2010	Xingtai	PHSN
04-1	2004	Qinhuangdao	PHGT	07-9	2007	Hengshui	FGQT	10-2	2010	Xingtai	THTT
04-2	2004	Unknown	MHGN	07-10	2007	Hengshui	PCNL	10-3	2010	Shijiazhuang	THHT
04-3	2004	Unknown	PHSN	07-11	2007	Hengshui	—	10-4	2010	Shijiazhuang	THTS
04-4	2004	Hengshui	PCQS	07-12	2007	Hengshui	PHSN	10-5	2010	Qinhuangdao	THKT
04-5	2004	Xingtai	THCT	07-13	2007	Hengshui	PHRT	10-6	2010	Shijiazhuang	THRT
04-6	2004	Baoding	PCJD	07-14	2007	Hengshui	PHHP	10-7	2010	Xingtai	THQS
04-7	2004	Langfang	DHQS	07-15	2007	Hengshui	THST	10-8	2010	Xingtai	PHHS
04-8	2004	Handan	PCBL	07-16	2007	Hengshui	TCLT	10-9	2010	Xingtai	THTS
04-9	2004	Qinhuangdao	PHRT	07-17	2007	Tangshan	THLT	10-10	2010	Qinhuangdao	PHRT
04-10	2004	Unknown	PHDN	07-18	2007	Tangshan	THCT	10-11	2010	Shijiazhuang	THTT
04-11	2004	Unknown	PCGT	07-19	2007	Tangshan	MHTS	10-12	2010	Shijiazhuang	THST
04-12	2004	Qinhuangdao	PHBL	07-20	2007	Hengshui	THHT	10-13	2010	Shijiazhuang	MHST
04-13	2004	Unknown	PCDN	07-21	2007	Chengde	PHRT	10-14	2010	Shijiazhuang	THTS
04-14	2004	Baoding	PHGP	07-22	2007	Chengde	THST	10-15	2010	Xingtai	THST
04-15	2004	Xingtai	THHP	07-23	2007	Shijiazhuang	PCTT	10-16	2010	Qinhuangdao	PCRT
04-16	2004	Xingtai	LBGL	07-24	2007	Shijiazhuang	SHRT	10-17	2010	Xingtai	THTP
04-17	2004	Unknown	PHGS	07-25	2007	Shijiazhuang	THTT	10-18	2010	Qinhuangdao	THTR
04-18	2004	Unknown	PHGR	07-26	2007	Xingtai	PHSN	10-19	2010	Qinhuangdao	THTS
04-19	2004	Baoding	PHQB	07-27	2007	Xingtai	PHRT	10-20	2010	Qinhuangdao	PCTT
04-20	2004	Unknown	PHQT	07-28	2007	Handan	PHJT	10-21	2010	Xingtai	THST
04-21	2004	Baoding	PGPN	07-29	2007	Handan	PHHP	10-22	2010	Xingtai	PHSS
04-22	2004	Baoding	NHJN	07-30	2007	Handan	—	10-23	2010	Qinhuangdao	PHST
04-23	2004	Handan	THQS	08-1	2008	Xingtai	THST	10-24	2010	Shijiazhuang	PHTN
04-24	2004	Baoding	PHQP	08-2	2008	Cangzhou	PHST	10-25	2010	Xingtai	THRT
04-25	2004	Baoding	FCJT	08-3	2008	Shijiazhuang	THLP	10-26	2010	Qinhuangdao	THFK
04-26	2004	Handan	PHJN	08-4	2008	Cangzhou	PHTT	10-27	2010	Shijiazhuang	THST
04-27	2004	Baoding	PCJN	08-5	2008	Cangzhou	PHBT	10-28	2010	Xingtai	THTT
05-1	2005	Baoding	FHHT	08-6	2008	Cangzhou	PHQP	10-29	2010	Shijiazhuang	THTN
05-2	2005	Baoding	TCTR	08-7	2008	Cangzhou	PHGS	Total number of isolates		247	
05-3	2005	Baoding	FHDQ	08-8	2008	Cangzhou	PCKT				

"—": The isolate was not identified successfully.
