# Supplementary material for: Virulence and molecular genetic diversity, variation, and evolution of the Puccinia triticina population in Hebei Province of China from 2001 to 2010
Source: Front Plant Sci. 2023 Mar 6;14:1095677. doi: 10.3389/fpls.2023.1095677 (PMC10025498; doi:10.3389/fpls.2023.1095677)
Supplement: Supplementary file 5 [file Table_2.pdf]

TABLE S2 The 21 pairs of EST-SSR primers used in this study.

**Primer name**	**Primer sequence (5'-3')**	**Primer name**	**Primer sequence (5'-3')**
PtSSR0083	F: ATGGATTTGGAGACCAGTCG<br>R: GTTGAAAGATCTGGGGGTGA	PtSSR0125	F: ATCGTGTCATGCAACCAAAA<br>R: AGAGAGGGACGTGAGGGATA
PtSSR6981	F: ACGTGGTGAGGTTTCTGCTC<br>R: TTCCGTTTTTGAAAGCAAGC	PtSSR5594	F: CGGACCAAACACAAAGGAAA<br>R: CCCTGCGTTTAACACCTTGT
PtSSR0019	F: GTTCGGATACCCCGTTTCTC<br>R: TTTGGAGCATGTTGTTTTGG	PtSSR0189	F: TCTCAACCAAAAATCAATCTACG<br>R: CTTCCACGAAGACGAAGCAC
PtSSR5649	F: CAGACGACCATCAACATTCG<br>R: CATGAACCAAACAAACAGCTTC	PtSSR0801	F: CAATGGTAGTGGCAAGCAAA<br>R: GCACCTCTCACGCTCTTAGC
PtSSR0085	F: CCAAAATTATCCCGCCCTAT<br>R: GCGAGGGGGTAGGAAGTAAT	PtSSR0481	F: CCACAATCCTCCGTTCTGAT<br>R: CGAAAGCAAAACACATGAGG
PtSSR6259	F: GTTCAACACATTGCGCTGTT<br>R: ATGGGTTGTGCAGATCGAGT	PtSSR0639	F: TCTCCGCCTACCAACACTG<br>R: AAAGGAGGGAGAGGGGAGG
PtSSR2948	F: CACACACCACACAAAACCAA<br>R: CCCAACAAGCTCGTGTCTTT	PtSSR3145	F: TAGGTGCGTGGTTTTCATCA<br>R: CAAATGAGAGCGACGAACAA
PtSSR0536	F: TGTTGCGAATTGATGGTACG<br>R: GAAGTTCTGCTCTGCTGTCG	PtSSR6542	F: TGTGATCTCGCCCGTACATA<br>R: TGGGAATGATGGACACACAC
PtSSR3233	F: GTAAGCTCGCTTTGGCTACG<br>R: TTTGGAGCATGTTGTTTCCA	PtSSR0182	F: CGAATCCCTTGTCTTTTGCT<br>R: TGTAGAGAGCGGGAGAAGAAA
PtSSR6863	F: TAGATGGGCACACAACCAAA<br>R: AAGCAAAGTGCAAGGAGCAT	PtSSR6386	F: AATGAGGTGACTCGGATGGA<br>R: GAAGAAGGCGAAGTTGTTGC
PtSSR0243	F: CTCACTCGCTCGCTTGTTCT<br>R: GACGAAAAGATCGGGTTTGA		
